# Impact of a Health Research Training Program on Patient and Community Partners, and Researchers: A Qualitative Evaluation

**DOI:** 10.1111/hex.70731

**Published:** 2026-06-26

**Authors:** Sadia Ahmed, Ingrid Nielssen, Iqmat Iyiola, Paul Fairie, Sandra Munro, Maria J. Santana

**Affiliations:** ^1^ Alberta Strategy for Patient Oriented Research University of Calgary Calgary AB Canada; ^2^ Department of Community Health Sciences University of Calgary Calgary AB Canada; ^3^ Faculty of Agricultural, Life, and Environmental Sciences University of Alberta Edmonton AB Canada; ^4^ Department of Pediatrics, Cumming School of Medicine and Department of Community Health Sciences University of Calgary Calgary AB Canada

**Keywords:** impact evaluation, patient partner training, patient‐oriented research, training programme

## Abstract

**Background:**

Building capacity through training and education is recommended to support meaningful engagement of patient partners in health research. The Patient and Community Engagement Research (PaCER) programme is a 12‐month initiative that enables patients and community members to co‐design and conduct qualitative health research projects. Learners are sponsored by clinician researchers, community organisations, and/or jurisdictional health organisations. This study aimed to assess the experiences and impact of the PaCER programme on: (1) past learners' personal development and engagement in health research; and (2) sponsors' research programmes.

**Methods:**

We conducted a qualitative impact evaluation involving past learners (alumni) and sponsors of the PaCER programme from 2019 to 2024. A purposive sampling strategy was used, whereby selected alumni and sponsors from the 2019–2024 cohorts were invited to participate in an online semi‐structured interview. Sponsors and alumni with diverse characteristics, including variation in age, ethnicity, educational attainment, and geographic location were invited. The interview guide included questions on the experiences in the PaCER programme, post‐programme research activities, and perceptions of the programme's impact. Data were analysed using a reflexive thematic analysis approach.

**Results:**

Eighteen participants (12 PaCER alumni and 6 sponsors) were interviewed, and four main themes were identified: (1) seeing the value of the training programme; (2) Diverse people and groups working together; (3) Learner readiness and capacity; (4) Academic opportunities for learners and career growth. Alumni described significant personal impact, empowerment, academic contributions, and increased access to meaningful patient partner opportunities. Alumni reported co‐authoring publications and presenting their work at academic conferences and to various stakeholders, including health system leadership. Many alumni had no prior experience with qualitative participatory research, and participation in the programme supported the development of these research skills. Sponsors also noted impacts on their research programmes, such as securing additional funding informed by PaCER project findings.

**Conclusion:**

The findings demonstrate that structured, experiential training for patients and community members supports meaningful engagement in health research, contributes to sustained capacity development, and positively influences the patient‐oriented research landscape.

**Patient or Public Contribution:**

Patient partners were involved in the design and conduct of the study (data collection), analysis and interpretation of the data, and in the preparation of this manuscript.

## 1 Background

As active and equal members of research teams, patient partners contribute their unique lived‐experience expertise to inform research priorities, study design, data collection, data analysis, and knowledge dissemination. To support meaningful engagement of patient partners, building capacity including training and education are recommended [1]. However, as identified by the Canadian Institutes of Health Research (CIHR) Strategy for Patient‐Oriented Research Capacity Development Framework, limited engagement of patient partners presents a key challenge to translating health research findings into clinical practice [1]. In response to this challenge, several courses and online modules have been developed to support education in patient‐oriented research (POR) for clinicians, researchers, trainees, patient partners, and other interest holders [2, 3, 4]. Examples include the self‐paced e‐learning modules *Patient Engagement 101: A Patient‐Oriented Research Curriculum in Child Health* [3] and the Alberta Strategy for Patient‐Oriented Research (AbSPORU) *Introduction to Patient Engagement in Patient‐Oriented Research* [2].

Instructional courses provide another form of delivery for patient‐oriented research training. An innovative programme for patient partners is the University of Calgary's Patient and Community Engagement Research (PaCER) programme [5]. PaCER is a certificate programme that teaches qualitative health research methodology and skills to teams of diverse, sponsored learners, making health research methods more accessible to the public [5]. The programme is fully funded through sponsorship, ensuring no cost to participating learners. Sponsors include academic and clinician researchers, community organisations and/or jurisdictional health organisations. The value and impact of the PaCER programme has been recognised at regional, national, and international levels [6, 7]. However, since an outcomes mapping evaluation from 2011 to 2013, no formal long‐term evaluation has been conducted to examine the perspectives of sponsors or PaCER alumni [7]. Since this period, the programme has undergone significant modifications, including a transition from in‐person to online delivery and the implementation of targeted recruitment strategies to engage patients and community members from diverse socio‐cultural, academic, professional and health research and health backgrounds. Understanding the impact of the PaCER programme on both sponsors and learners is therefore critical to advancing patient‐oriented research.

This paper presents a qualitative impact evaluation of the PaCER programme on sponsors and alumni (past learners) covering the period from 2019 to 2024. The evaluation aimed to explore participants' (sponsors and alumni's) experiences of the programme and to assess programme impacts on: 1) alumni's personal development, and engagement in health services research; and 2) sponsors' research programmes including national learning health systems' programmes.

### 1.1 Context: Patient and Community Engagement in Research (PaCER) Programme

Patient and Community Engagement in Research (PaCER) is a three‐course certificate programme delivered online through University of Calgary Continuing Education with academic and scientific support and oversight provided by the Alberta SPOR SUPPORT Unit (AbSPORU) Patient Engagement Team [5].

The 12‐month programme teaches qualitative health research methodology and skills to teams of diverse, sponsored learners who then apply these skills to develop, design, and conduct a qualitative health research project about a health condition relevant to them and their sponsor. One of the requirements to join the programme is that students need access to a computer and reliable internet.

PaCER projects are sponsored by academic and clinician researchers, community organisations and/or jurisdictional health organisations who are interested in developing research capacity in their patient and community partners as well as gaining peer‐to‐peer patient experience evidence to help inform their programmes of research. The PaCER sponsors provide a research topic for their team, provide specific contextual information, support recruitment of students, and receive a final report from the learners at the end of the program. Sponsors also provide funding for around 6‐8 students per project.

In total, there are 160 h of project‐based learning distributed through 1 year in three distinctives courses [5]. In Course 1, students gain an understanding of health research methodology and skills, including literature reviews, qualitative data collection, thematic analysis, and research ethics. Student teams then apply these skills in Course 2 to co‐design a qualitative patient‐experience study protocol about a topic relevant to them and their sponsor. This protocol is submitted for institutional research ethics board approval and in Course 3 the teams carry out their studies, finishing with a final report to their sponsors. Study findings are often shared as publications in peer‐reviewed journals, at conferences. and increasingly inform subsequent research phases and studies. Sometimes students choose not to continue with the program, especially during Course 2. New team members join those projects instead to support the project moving forward. From 2019 to 2024, 78% of students completed the PaCER program. The PaCER program provides them with access to downloadable Microsoft Office and they gain basic skills in Word, Excel and PowerPoint throughout the program if they don't already have this and learn to work on shared documents. Other access to software includes Zoom, Outlook and Qualtrics.

## 2 Methods

### 2.1 Study Design

We conducted a qualitative impact evaluation of the PaCER program on alumni and sponsors for the period 2019–2024. Impact evaluation examines the effects of an intervention, which may include a discrete project, a program, a set of activities, or a policy [8]. A qualitative approach was selected to obtain in‐depth insights into the experiences and perspectives of PaCER alumni and sponsors. This study is reported in accordance with the Consolidated Criteria for Reporting Qualitative Research (COREQ) checklist and GRIPP2 checklist (Appendix A) [9]. The study has been approved by the University of Calgary Health Research Ethics Board (REB25‐0590).

### 2.2 Evaluation Model

A logic model was developed to articulate the program inputs, activities, and short‐, medium‐, and long‐term outcomes (Appendix B). This model informed the planning of the evaluation and the development of the interview guide. In addition, the Kirkpatrick model was used to guide the evaluation design, with a specific focus on Level 4 (Results) [10]. The Kirkpatrick model is a recognised method for evaluating the results of training and learning programs [10]. The model is made up of four criteria: reaction (level 1), learning (level 2), behaviour (level 3), and results (level 4) [10]. Levels 1‐3 tend to focus on immediate reactions, learning acquired, and behavioural changes following the training program. For level 4, a longer timeframe is usually required for the evaluation to track outcomes/results following a training program.

### 2.3 Participant Recruitment

A purposive sampling strategy was employed, whereby selected alumni and sponsors from the 2019–2024 cohorts were invited to participate in an online semi‐structured interview. We compiled a list of sponsors and alumni who participated in the PaCER program between 2019 and 2024. Recruitment was further refined by an intentional effort to include PaCER alumni from diverse backgrounds, including variation in age, ethnicity, educational attainment, and geographic location, as well as PaCER teams. Eligible participants were emailed an information and consent form and were provided the option to provide written and/or verbal informed consent. PaCER alumni were offered a CAD $25 electronic gift card in recognition of their participation.

### 2.4 Reflexivity and Research Team Positionality

The team was ethnically and culturally diverse and included members of different ages, genders, professional backgrounds, and roles. These varied characteristics and experiences contributed to multiple analytic perspectives during interpretation of the data. The research team consisted of three academic researchers, two patient partners, and a patient engagement coordinator, who all had an established relationship with the programme and were familiar with its aims, structure, and pedagogical approach. This insider positioning supported contextual understanding of the study setting but required reflexive consideration of how researchers' characteristics, roles, and assumptions may have influenced data collection and interpretation.

Two of the academic researchers (PhD, background in Health Services and Patient‐Oriented Research) have provided scientific and academic oversight to the PaCER program since 2019, and the third academic researcher (MSc, background in Health Services Research), who led this evaluation, has provided guidance and support for the program's research ethics board applications. One patient partner completed the PaCER program before 2019 and continues to provide mentorship and recruitment. Interviews were conducted primarily by the second patient partner, who was a graduate of the PaCER program and had prior experience as a patient partner in health services research and training in qualitative methods. The interviewer was pursuing undergraduate studies in Life Sciences at the time of the study. The interviewer had no direct prior working relationships with participants, aside from overlapping participation in the PaCER program, and had not collaborated with participants on the same projects. This positioning supported rapport while minimising role‐related power differentials. Two interviews (one with a sponsor and one with a PaCER alumni) was conducted by the academic researcher, rather than the patient partner to avoid any conflict of interest.

The Patient Engagement Coordinator served as an instructor in the PaCER program during part of the evaluation period (2023–2024). This individual had direct involvement in program delivery and interaction with learners, which provided insight into participant experiences and learning processes. To address potential power imbalances and the influence of this role on the research, the instructor did not conduct interviews and was not involved in initial transcript coding. The instructor's contributions were limited to later stages of interpretation through team‐based discussions.

Reflexivity was addressed through a team‐based analytic approach, in which two research team members engaged in coding and interpretation. Regular analytic meetings with other team members were held to compare interpretations, discuss discrepancies, and critically examine identified themes. Reflexive discussions and memo‐writing were used throughout data collection and analysis to document researchers' assumptions, positional influences, and analytic decisions. These strategies enhanced transparency and supported the credibility, dependability, and confirmability of the findings.

### 2.5 Data Collection and Analysis

The semi‐structured interview guides were co‐developed by members of the research team and included questions related to participants' experiences in the PaCER program, research activities following program completion, and perceptions of the programme's impact on their professional trajectories. Two separate guides were developed (one for PaCER alumni and one for sponsors) (Appendix C).

All interviews were conducted online via Zoom between May and July 2025, as PaCER alumni and sponsors were geographically dispersed across Canada. Interview durations ranged from 17 to 66 min. Audio recordings were transcribed verbatim using Zoom transcription. Transcripts were subsequently reviewed by the patient partner to verify accuracy and were deidentified to ensure participant confidentiality.

### 2.6 Data Analysis

All interview transcripts were analysed using a reflexive thematic analysis approach. The systematic and iterative process of coding included the following steps as identified by Braun & Clarke [11]. Thematic Analysis steps included: 1) familiarising yourself with the data. In this step the patient partner researcher listened to the audio recordings and transcribed the data for errors after Zoom transcription. The steps afterward were 2) Generate initial codes. The two coders used both deductive and inductive coding strategies in Excel. The patient partner researcher independently coded the transcripts inductively, and then the health service researcher reviewed the codes and made notes on codes that needed further discussion. After inductively coding, both coders created a codebook to guide deductive coding for the rest of the transcripts. In the third step of generating initial themes, codes were clustered together into a tentative list of themes. Steps afterwards were (4) Developing and reviewing themes, (5) Refining, defining and naming themes, and finally (6) writing up. Strategies were used to enhance the credibility of our work such as member checking (where the interviewer would confirm responses of the participants and ask for clarification).

We also engaged in peer debriefing sessions between the two coders to discuss the codes identified, our interpretations, and organising a codebook that aligned with our interpretations. We maintained an audit trail to enhance dependability‐ we documented the entire coding process, decisions, and findings from the project. Our data collection was focused on identifying meaningful insights from our participants. As recommended by the Braun & Clarke method [12], data saturation was not the focus for this study. After speaking with the alumni and sponsors from each of the PaCER projects between 2019 and 2024, we identified a comprehensive list of themes that align with the objectives. Rather than achieving thematic saturation, the themes were sufficiently well developed in the context of our research question after speaking with 18 participants [12].

## 3 Results

We invited 13 PaCER alumni and 9 sponsors to participate, of which 12 PaCER alumni and 6 sponsors agreed to participate. Out of the 12 PaCER alumni, the year of graduation varied from 2020 to 2024. Majority of the participants identified as women (72%), the alumni ranged between from 18 to older than 60 years. Participants cultural backgrounds included Caucasian (8/18, 44%), Latin American (4/18, 22%), South Asian (2/18, 11%), Indigenous (2/18, 11%), Chinese (1/18, 6%), and Azerbaijani (1/18, 6%). A summary of the demographic characteristics of participants is below in Table 1.

**Table 1:** Demographic characteristics of participants, *N* = 18 (alumni, 12 and sponsors, 6).

	Alumni total *n* (%)	Sponsors total *n* (%)
Age range (years)		
18‐29	5 (42)	0
30‐39	3 (25)	1 (17)
40‐49	2 (17)	2 (33)
50‐59	0	2 (33)
60+	2 (17)	0
Gender		
Man	2 (17)	2 (33)
Woman	9 (75)	4 (67)
Non‐binary	1 (8)	0
Education		
High School	3 (25)	0
Undergraduate	7 (58)	0
Post‐graduate	2 (17)	6 (100)
Cultural background		
Caucasian	6 (50)	2 (33)
Latin American	3 (25)	1 (17)
South Asian	1 (8)	1 (17)
Status Indian First Nation/Indigenous	1 (8)	1 (17)
Chinese	0	1 (17)
Azerbaijani	1 (8)	0

The overall aim of conducting the interviews was to understand the experiences of taking the program, and how the PaCER program has impacted the alumni's personal growth, and engagement in health and health systems research. We also wanted to understand how the PaCER program has impacted the sponsors' research programs.

Through thematic analysis, we identified four main themes related to the evaluation including:

1. Seeing the value of the training program;
2. Diverse people and groups working together;
3. Learner readiness and capacity;
4. Academic opportunities for learners and career growth

The themes are described below and presented in the Table 2 with illustrative quotes. Table 3 mapped the findings in alignment with the objectives of the evaluation.

**Table 2:** Main themes with supporting quotes.

Theme	Description	Quotes
Seeing the value of the training program Sponsor perspectives	Perspectives from sponsors on their motivations for sponsoring a PaCER cohort. Motivations from sponsors included health system funding and seeing the value of engaging patient and community members in research.	‘I was incredibly fortunate that the funding was made available by the [strategic clinical network] SCN at that time. And they could see the value.’ S6 ‘we had made a commitment to sponsor Indigenous PaCERs to do work in cancer prevention.’ S1 ‘[PaCER]it's really innovative, so I thought, ‘oh, here's a perfect match for their patient engagement work', and then the Engagement work we started… we got the funding… and planned, uh, began the PaCER cohort in 2023.’ S2 ‘I saw this disconnect between what we are focusing on as******* clinicians and researchers, and what families want changed. So I thought the best way to ask families that are from diverse backgrounds what it is that is important to them…was to have…. people that have had lived experience. From all diverse backgrounds. Um, speak to this, and ask people about these questions. So rather than having a graduate student who is, again, one of us.’ S4
PaCER Alumni Perspective	Motivations from the PaCER alumni included opportunity for personal and professional development, and contributing to health research and healthcare	‘it was basically to… to give myself a better foundation on… participating in research, and …I guess that the other important thing here is that…I was beginning… this is a brand new journey for me, right? So… as I was looking through these…opportunities. I realised that several of them were looking for people that had… lived experience, and…a PaCER background or a PaCER certificate.’ P1 ‘And so, I found that research work in fact, informed the health system and at large policy. And so that's something that was… very, very rewarding for me.’ P12 ‘I always wanted to be a part of the research world, but I didn't really know where to start with that. And when I joined PaCER, honestly speaking, I didn't even know where it's going to lead me. I just thought, you know, it's just a nice experience for me to be doing something in Canada, just starting with that. And that's how it started. But it led me to such beautiful places… it honestly just opened up the research world for me.’ P5
Diverse people and groups working together	Both alumni and sponsors described the positives of having a diversity of learners working together on a PaCER project. Some alumni talked about the opportunity to learn from their peers was valuable. Participants mentioned the ability to manage group dynamics and resolve group conflicts in a respectful way was crucial for a group's success.	‘And I enjoyed learning also from the other participants in the other projects, it was…a great opportunity to learn from them and hear from them. On our… weekly class…and see different perspectives.’ P8 ‘I think it was really cool to get to work in a group, like a smaller group like that, and… kind of figure out how to manage those group by… group dynamics a bit, because it… it is an experience you have to learn working on a research team’ P2 ‘We didn't have to do anything necessarily with the research ethics application, which was really great. I think that's where you [AbSPORU] came in because that was just a lot of work and you know, we were novices …So that was a really great help that was kind of like submitted for us on our behalf, but we didn't have to really take care of any of that thought. That was really great.’ P4 ‘One of the people…in my group was an excellent facilitator, and I've learned a lot just from watching and working with her, and I've worked with her on another… two other projects as well now.’ P6
Learner readiness and capacity	Alumni and sponsors highlighted a few key challenges of the PACER program. These included student readiness, program commitment, and other barriers to engagement.	‘I found that we were just not prepared for what we were undertaking. I recall some of the teammates saying that ‘we did not realise the time commitment that would be put in.’ P12 ‘It was pretty overwhelming at times, like, it could be intense. Sometimes there could be personality clashes within it, and… yeah. So, I'd say the knowledge was good. Gained a lot of insight from it, but the experience was incredibly stressful.’ P9 I see this across, like, the engagement spaces that I work in, is, like, it attracts a lot of quite older people that are retired. Uh… entice them to, like, want to be engaged…I would like to see it [PaCER] expanded.’ P2
Academic opportunities for learners and career growth	Several strengths were highlighted that included the academic value of the program, enhancement of employment opportunities, personal and professional growth, and value of strong academic leadership.	‘I think it was a very structured program with a really good blend of didactic and interactive sessions. There were enough student supports throughout the program to make sure that you know, everyone felt like they had the tools they needed to complete the project. My team especially, I felt like our sponsor and our kind of group leaders were really good at ensuring that the project didn't end at the end of the PaCER program, there was enough opportunities for knowledge mobilisation. And then, yeah, at the end, it was really good, because we stayed connected through the Facebook group and stuff like that.’ P11 ‘So many of us are making a difference. I think that is an added value, feeling that sense of community and belonging.’ P7 ‘just gave me an opportunity to… more formally learn a little bit more about the research process, the entire research process itself’ P6 ‘Everything that we were learning, we were putting into practice throughout the program.’ P8 ‘Sometimes it's nice for, like, the academics or researchers to see, oh, I've, like you said, done the formal training, too’ P6
Subtheme: impacts of the program	Participants expressed the impacts of the program include academic outputs and knowledge dissemination opportunities, further funding opportunities, and impacts on personal and professional growth.	‘the ultimate goal with the videos, which the PaCER program has helped create, you know, more effective videos…to disseminate them, …through partners that we're seeking with nonprofit charitable organisations. We've let the Public Health Agency of Canada know about them’ S2 ‘I think it made us more competitive for funding, which we… As I said, we got kind of two rounds of funding, so it helped us expand from a smaller scale to a bigger scale project.’ S2 ‘it opened up so many doors for me with connections and my research experience and potentials from like my job and and everything’ P4 ‘…the work has received national recognition and has actually a lot of that work helped us get a grant from the government to sponsor a virtual chronic pain program. And a lot of the evidence came from the PaCER work. And the PaCERs continue to help support that work. So we got a $1.5 million grant to build a virtual pain program with peer support, which was some of the finding that came out of the PaCER program. And that grant is underway today.’ S1 ‘this year we participated in… in a few conferences, too… We did the presentation. And we… we just submitted the manuscript to the… to the journal, to the Frontier Journal, so hopefully it's going to be accepted.’ P5

**Table 3:** Mapping evaluation objectives to findings.

	Objective 1 Alumni personal development and engagement in health research	Objective 2 Sponsors' research programs
1. Seeing the value of the training program	Provided insight into intrinsic drivers (e.g., desire to help others, use lived experience, interest in research, interest in capacity building).	Provided insight into Sponsors' motivations such as availability of funding and seeing the value of meaningful engagement of patients.
2. Diverse people and groups working together	Development of collaborative skills and peer learning among alumni with varied backgrounds and health conditions, enriched personal development and broadened perspectives.	The role of sponsors was to provide guidance and mentorship for the teams to work together. This included guidance on the research topic, background, and design of the project.
3. Learner readiness and capacity	Highlighted challenges that alumni faced while in the program such as readiness, program intensity, and digital literacy.	Some sponsors also highlighted understanding of expectations for both sponsors and students as a challenge.
4. Academic opportunities for learners and career growth	Demonstrated program efficacy in building patient research capacity, teaching qualitative methods, and connecting graduates to a professional network for future engagement opportunities.	The program provided evidence of the value of the patient‐led, peer‐to‐peer research model, which can lead to actionable recommendations for policy and practice change that incorporate the patient voice.

*Note:* The table shows the alignment of findings to the overarching goals of the PaCER program evaluation. The findings related to alumni experience align with objective 1, while those related to project outcomes and patient engagement in health research align to both objectives, as the development of patient researchers inherently benefits research programs.

### 3.1 Theme 1: Seeing the Value of the Training Program

This theme described the perspectives of sponsors and their motivations for sponsoring a cohort of PaCER students, as well as the perspectives of PaCER alumni on their motivations for joining PaCER.

### 3.2 From the Sponsor Perspectives

The sponsors described receiving financial support from health system funders for patient‐led POR projects, for health issues and conditions that were a priority in the healthcare system. Those sponsors that received funding from the health system mentioned it was important to offer training for patient and community members through PaCER so that the patient and community members could actively engage in health system projects.

My role as a sponsor not only was to allow PaCER development to help advance the work that I was doing, but to also help advance… patient‐oriented research across the 16 SCNs [Strategic Clinical Networks], so… every network, all 16 SCNs had PaCERs, trained PaCERs who were working with the research community within that network. *S1*

Sponsors mentioned seeing the value of providing training to patient and community partners so that they can meaningfully engage in health research. Sponsors spoke about having available grant funding and furthering their research through additional phases.

I'll say it was building a talent pool of… of researchers with useful skills that led to it. *S3*

I think that we wanted to have patient input in research. And to have their voices heard, and… having them trained in research methodology does help. And so that they can actually feel more comfortable…in their participation. *S5*

### 3.3 From the PaCER Alumni Perspectives

PaCER alumni described their motivations for taking the PaCER program was for (1) personal and professional development; and (2) contributing to health research and healthcare.

Most alumni identified academic motivations for why they enroled in the PaCER program. This ranged from the value of obtaining a certification, the academic recognition of the PaCER program, and using the knowledge gained to make health research more accessible. Other motivators included building their own patient partner network, capacity building and empowerment, and professional development. Alumni valued the opportunity to contribute to the advancement of health research and policy.

One PaCER alum spoke about the opportunity to be a learner within the program:

this is such a great opportunity to be a learner, because in the future, I don't think I'll get those in my academic career or my professional career. No one's gonna take me under their wing and show me and teach me and support me. They're just gonna expect me to know'. And I think that was really important, that I felt really supported, and…I'll never get an opportunity like this again. *P10*

Another PaCER alum mentioned wanting to contribute to the advancement of health research and to support others with similar lived experiences:

I never knew… of a program that actually involves patients with lived experience. And I thought that that is… amazing. It's a huge, um, milestone for research. So, I wanted to be part of it. That's the first thing. Uh, the second thing is that… I wanted to help others… like me. Other people with difficult pregnancies that were struggling, I feel that a lot of people don't know how research really works, but when research is truly patient‐oriented, it's meant to serve the community, and that is my way to give back. Yeah, so that was my motivation. *P7*

### 3.4 Theme 2: Diverse People and Groups Working Together

Participants described the experience of working on a PaCER project to be unique, especially as it brought together individuals of many diverse skill sets and experiences. Both alumni and sponsors described the positives of having a diversity of learners working together on a PaCER project. Some alumni talked about the opportunity to learn from their peers was valuable.

One person mentioned how all team members opinions and views were valued:

There were people with a clinical background, and the people with no clinical background in that group…Nobody was ever dismissed. Every opinion was listened to, was considered, was explained. Every question was answered. It was a beautiful experience to see people from so many diverse backgrounds, and backgrounds not just in terms of ethnicity and race and religion, but also in terms of culture and geography, um, and lived experience that were working together. Collectively, consistently overall. Over a 10‐month period. It was beautiful. *S4*

Another alum spoke about learning from not only their sponsor research teams, but also their peers in the program:

I think just learning not only from, the people who supported us that have knowledge, but learning from each other, questioning each other in a healthy and safe and educational way felt really, really comfortable, and it challenged the way I think. *P10*

Participants mentioned the ability to manage group dynamics and resolve group conflicts in a respectful way was crucial for a group's success. Alumni and sponsors describe how they managed challenging group dynamics that occurred in their teams and the roles of the sponsor research team and program administrators (cohort liaison, course instructor, scientific director, etc.) in supporting students. Some of the group dynamics resulted from challenges such as a few PaCER alumni mentioned the division of work was not always equal.

One alum mentioned the level of support within the program:

There's a great, you know, source of information for us. So we had a lot of different separate team meetings with [our sponsor] and [our team support]. And [our course instructor] was really great too. She was involved a lot with if we had questions and would pop in and stuff. *P4*

Another alum spoke about the disappointment of some team members not contributing:

And so some…would show up and not say anything, and stuff like that was a little difficult… it would have been nice for everyone to have that same level of, maybe, commitment. *P10*

### 3.5 Theme 3: Learner Readiness and Capacity

Both alumni and sponsors highlighted some few key challenges they experienced in completing the program. Some sponsors and alumni mentioned student readiness and understanding of expectations were challenges for some of the alumni. Some students struggled with computer literacy and technological skills or underestimating the level of work required. Lack of readiness for the students also impacted their stress and how they managed the course requirements. One person also explained having lower capacity to contribute to their project due to their health condition.

One learner described how overwhelming the program could be:

It was pretty overwhelming at times, like, it could be intense. Sometimes there could be personality clashes within it, and… yeah. So, I'd say the knowledge was good. Gained a lot of insight from it, but the experience was incredibly stressful. *P9*

One sponsor spoke about changes that happened to support students in the program:

Some of the students had a lot of trouble getting onto [Desire2Learn‐ online learning platform] D2L and their computer skills weren't as sophisticated because some of these people weren't university students. So we learned through that process that we needed to provide different supports for those students. So two things happened as a consequence: one, we did build in a… tutorial on how to do it prior to the class starting. And secondly, we changed in our recruitment process for those becoming PaCERs. One of the things we assessed was their ability, their computer skills. Did they have you know, proper equipment at home? Were they able to troubleshoot the internet, etc? *S1*

### 3.6 Theme 4: Academic Opportunities for Learners and Career Growth

Most participants described the value of the PaCER program was in its academic rigour, enhancement of employment opportunities, personal and professional growth, and value of strong academic leadership. Participants described the value of the program in being offered through an academic institution. Some alumni also explained how they were perceived more favourably in job prospects when pursuing opportunities.

One sponsor highlighted academic rigour of PaCER:

I found out about PaCER… And I realised that that was the answer to what I was looking for, because that's a group that has done this before, it's not an experiment, they've done it, and they've done it in a systematic manner, they've done it through a university. There is a very structured approach to how they do it. *S4*

One alum mentioned the growth in opportunities following PaCER:

Learning skills about the research process helped me get jobs, you know, it was really good to talk about it during job interviews or add it to my CV. *P11*

### 3.7 Subtheme: Impacts of the Program

Participants also spoke of how the PaCER program impacted their personal growth. For many of the alumni, they did not have prior qualitative participatory research experience. Therefore, the program helped them gain research and organisation skills, as well as developing collaborative skills to work on projects.

I succeeded at…working on my communication…and cognitive capacity. And my confidence with those. *P3*

One alum mentioned how PaCER made working in research more accessible for those without higher education:

my perception of research was you have to be…. you had to have, like, a doctorate or a PhD in this high level of education, and things like that, and I never thought I could be a part of a project like that. And so, for it to be a student cohort that had a support team that was really there for us, like, none of this would have happened without the support team. It was just really empowering that someone like me could even be part of that, and I think it changed the way I view things… it was eye‐opening that I could challenge myself. *P10*

Some of the impacts of the PaCER program mentioned by both PaCER alumni and sponsors are the academic outputs and knowledge dissemination opportunities resulting from PaCER. Participants spoke about the opportunity to co‐author publications from their PaCER projects, present their project at academic conferences and with various interest holders such as health systems leadership and other partners. Some sponsors also discussed impact on their research agendas like securing further funding based on results of PaCER project.

One alum spoke about a further academic opportunity following PaCER:

I got an opportunity that I wouldn't have without PaCER which was a, um, I'm on [an] adjudication panel for grants. *P3*

## 4 Discussion

This qualitative impact evaluation identified four key themes describing participants' motivations for enroling in the PaCER program, their experiences within the program, and the perceived impacts on both alumni and sponsors. PaCER alumni commonly described motivations related to personal and professional development and a desire to contribute meaningfully to health research and healthcare improvement. Sponsors, in contrast, emphasised their motivation to support training opportunities that would enable patient partners to engage meaningfully in patient‐oriented research projects.

Importantly, participants' motivations and expectations aligned closely with the reported impacts of the program. Alumni described outcomes that reflected their initial goals, including enhanced research skills, increased confidence, and sustained engagement in health and health systems research. Sponsors reported that the program not only enabled patient partners to contribute substantively to research projects but also generated tangible benefits for their research programs, including successful grant funding, presentations at national and international conferences, strengthened partnerships with interest holders, and co‐authorship of peer‐reviewed publications [13, 14, 15]. Additionally, reported strengths of the PaCER program—such as enhanced employment opportunities for alumni and academic recognition of the program—further demonstrated its impact. Collectively, these findings align with Level 4 (Results) of the Kirkpatrick evaluation model, which focuses on measuring the direct outcomes of a program.

The PaCER program offers a distinct model for capacity development in POR, particularly for patients and community members who are new to community‐based participatory and POR. Through its emphasis on experiential, peer‐led research, the program provides participants with opportunities to develop research skills through active engagement in a patient‐led qualitative project. The program's 12‐month duration allows for sustained learning and application, distinguishing it from shorter‐term training initiatives. Furthermore, the online delivery format enhances accessibility for participants across Canada and beyond, while retaining flexibility for projects to include in‐person components when feasible. Some challenges reported by alumni and sponsors highlight the intensive nature of the programme, such as the time commitment required, managing group dynamics, and readiness. Therefore, those who join the progrm will need to consider their capacity and readiness to contribute to a health research project.

Several POR training programs described in the literature differ from PaCER in scope, duration, and pedagogical approach. For example, the Partners in Research course is a 2‐month online program that uses webinars, quizzes, assignments, and group coaching to introduce patient‐oriented research principles [16]. Similarly, the Family Engagement in Research (FER) course is a 10‐week online program focused on building partnerships between families and researchers in child health and neurodevelopmental disability research, with participants collaborating on the development of a shared resource [17]. While these programs provide valuable foundational knowledge, their shorter duration and limited experiential components contrast with the sustained, project‐based learning central to the PaCER model.

Other training initiatives, such as the Community Research Training Program described by Cunningham‐Erves et al., [18] provide in‐person training for community members and organisations but do not require participants to complete a research project. Experiential components are present in programs such as the Partners in Education Evaluation and Research Program; however, participation is restricted to individuals embedded within community organisations [16]. In contrast, PaCER is specifically designed for patients and community members and emphasises patient‐led research as a core feature. Ethics‐focused certification programs for community research partners further differ in scope, as they primarily address research ethics, consent, and confidentiality rather than broader research design and conduct of research projects [19].

A key contribution of this study is its examination of longer‐term impacts of a comprehensive patient‐oriented research training program. Participants completed the PaCER program between one and 5 years prior to the evaluation, allowing for reflection on sustained outcomes beyond immediate post‐training effects. In contrast, evaluations of other training programs have largely focused on short‐term impacts. For example, Courvoisier et al. [16] evaluated the Partners in Research course at baseline, immediately post‐course, and 6 months post‐course, finding improvements in knowledge, confidence, and intentions related to patient‐oriented research. Similarly, evaluations of the FER course were conducted immediately following course completion [17]. Cunningham‐Erves et al. [18] conducted a 1‐year post‐training evaluation, in which participants reported increased confidence in pursuing active research roles but expressed a desire for longer training to support application of learning. Consistent with these findings, PaCER alumni in the present study emphasised the value of applying knowledge through the program's project‐based component and reported meaningful opportunities to extend their work and engagement beyond program completion.

A notable strength of this study is its POR approach. A PaCER alumna served as a co‐author and conducted most interviews, contributing to data analysis and interpretation in collaboration with a health services researcher. This approach supported rapport with participants and ensured that interpretations were grounded in lived experience. However, several limitations should be acknowledged. The study focused exclusively on alumni and sponsors who completed the PaCER program between 2019 and 2024, reflecting the period during which AbSPORU assumed academic oversight and implemented substantive changes to program structure and delivery, including the transition to online delivery. Consequently, perspectives from alumni who completed the program prior to 2019 were not included. Additionally, eligibility criteria prioritised alumni known to have continued engagement in POR, potentially excluding perspectives from individuals who did not complete the program or did not continue research involvement.

Future evaluations should consider examining the long‐term impacts of the PaCER program across its full history since inception in 2012 and include a broader range of alumni experiences. Such work would provide a more comprehensive understanding of program outcomes and inform continued refinement of patient‐oriented research training initiatives.

In conclusion, this qualitative impact evaluation provided in‐depth insight into the experiences and perceived impacts of the PaCER program from the perspectives of both alumni and sponsors. Findings demonstrated that structured, experiential training for patients and community members can support meaningful engagement in health research, contribute to sustained capacity development, and positively influence the patient‐oriented research landscape.

## Author Contributions

**Sadia Ahmed:** conceptualisation, investigation, writing – original draft, methodology, validation, visualisation, formal analysis, project administration, data curation, writing – review and editing, supervision. **Ingrid Nielssen:** Conceptualisation, writing – review and editing, validation, formal analysis, resources, methodology. **Iqmat Iyiola:** investigation, writing – review and editing, methodology, validation, formal analysis, data curation. **Paul Fairie:** Conceptualisation, investigation, writing – review and editing, resources, formal analysis, methodology, validation. **Sandra Munro:** conceptualisation, investigation, writing – review and editing, formal analysis, validation, methodology. **Maria J. Santana:** conceptualisation, investigation, funding acquisition, writing – original draft, writing – review and editing, supervision, resources, project administration, formal analysis, validation, methodology.

## Ethics Statement

The study has been approved by the University of Calgary Conjoint Health Research Ethics Board (REB25‐0590). Participants provided informed consent to participate in this study.

## Conflicts of Interest

The authors declare no conflicts of interest.

## Supporting information


**Supporting File 1:** hex70731‐sup‐0001‐Appendix_B_Logic_Model.

**Supporting File 2:** hex70731‐sup‐0002‐Appendix_C_Interview_Guide_PaCER_Evaluation_3.

**Supporting File 3:** hex70731‐sup‐0003‐coding_tree_PaCER_Evaluation.

**Supporting File 4:** hex70731‐sup‐0004‐COREQ_PaCER_Impact.

**Supporting File 5:** hex70731‐sup‐0005‐GRIPP2_Short_form.

## Data Availability

All data generated or analysed during this study are included in this published article [and its supplementary information files]. The data that support the findings of this study are available in the supporting material of this article.
