# Supplementary material for: Impact of a Health Research Training Program on Patient and Community Partners, and Researchers: A Qualitative Evaluation
Source: Health Expect. 2026 Jun 26;29(4):e70731. doi: 10.1111/hex.70731 (PMC13307345; doi:10.1111/hex.70731)
Supplement: Supplementary file 1 — Supporting File 1: hex70731‐sup‐0001‐Appendix_B_Logic_Model. [file HEX-29-e70731-s004.docx]

**Objectives**

1. To understand how the PaCER program experience impacted PaCER alumni’s personal development and engagement in health and health systems research
2. To understand the impact that sponsoring a PaCER team has had on the sponsor’s program of research

**Methods**

**Logic Model for objective #1**

Assignments (e.g. proposal writing, preparing ethics documents)

Research Ethics Board Submission

Data collection and analysis

Report Writing

Research opportunities (conferences, presentations, workshops, grants, awards, publications, peer review)

Collaboration on Patient-Oriented Research Projects (as patient partners/co-researchers)

Academic endeavours (undergraduate, graduate school, medical school, etc.)

Employment opportunities (research, health system, patient and community organizations)

**Logic Model for Objective #2**

Research opportunities (conferences, presentations, workshops, awards, publications)

Conducting Patient-Oriented Research Projects (with patient partners/co-researchers)

Funding Grants received with inclusion of people with lived experience

Guiding PaCER students in the completion of their research projects

Supporting PaCER students in study outputs (report writing, presentations)
