# Supplementary material for: Impact of a Health Research Training Program on Patient and Community Partners, and Researchers: A Qualitative Evaluation
Source: Health Expect. 2026 Jun 26;29(4):e70731. doi: 10.1111/hex.70731 (PMC13307345; doi:10.1111/hex.70731)
Supplement: Supplementary file 2 — Supporting File 2: hex70731‐sup‐0002‐Appendix_C_Interview_Guide_PaCER_Evaluation_3. [file HEX-29-e70731-s005.docx]

**Interview Questions- PaCER Alumni**

1. Please tell me what year you took the PaCER program?
2. How does PaCER fit within your healthcare or research career journey?
   1. Prompt: Please briefly describe the health condition that motivated you to take part in the program.
3. What interested/motivated you to take part in the PaCER program?
4. Please tell me about what your overall PaCER experience was like?
5. How clear were the roles of those in the program?
   1. Prompts: Sponsor team, Instructor, yourself, and other team members.
6. Describe your research interests and research experience prior to taking the PaCER program.
7. Please tell me about the additional research work you have done to share your PaCER project.
   1. Prompts: conferences, publications, etc.
8. Describe your research engagement journey or additional research experience(s) you have had since taking the PaCER program.
   1. Prompts: conferences, publications, grants, awards, research positions/patient partner opportunities
9. Can you share the impacts taking the PaCER program has had on you?
   1. Prompts: personal, professional, etc.
10. Based on your PaCER experience, what would you suggest to improve the program?
    1. Prompts: the training, supports, etc.
11. Is there anything else that you would like to share?

Demographic Questions (if you are comfortable answering)

1. How would you describe your gender identity?
2. How would you describe which age range you were in when you first took PaCER?
   1. 18 years old – 29 years old
   2. 30 – 39 years old
   3. 40 – 49 years old
   4. 50 – 59 years old
   5. 60+ years old
3. How would you describe your educational background when you took PaCER?
4. How would you describe your educational background now?
5. How would you describe your ethnicity/cultural background?

**Interview Questions- PaCER Sponsor**

1. Please tell me which year(s) you’ve sponsored a PaCER cohort.
2. Have you sponsored more than one, or are you considering sponsoring more than one cohort?
3. What interested/motivated you in sponsoring a PaCER cohort?/sponsoring your first cohort?
   1. Prompts: team of students, your role
4. What motivated you to sponsor a second/multiple cohorts?
5. Please tell me about what your overall PaCER experience was like?
6. Once you heard from PaCER, tell me about contacting with and registering a cohort.
   1. Prompts: including contracts and supports available
7. How clear was the roles and responsibilities of each of the people involved in the process? This includes the Sponsor, PI, COntEd IT supports, Liaison, Instructor.
8. What has been your experience collaborating with patient and community partners prior to PaCER?
9. How does sponsoring a PaCER cohort support your program of research?
   1. Prompts: i.e. training people with lived experience to more meaningfully contribute; the results of the PaCER projects; learnings from PaCER project design, methods, processes; participant recruitment
10. Please tell me about the opportunities you have sought or received due to PaCER?
    1. E.g. conferences, publications, research positions, grants, awards, etc.
11. From your experience, what has been the overall impact after sponsoring a PaCER cohort?
    1. Prompt: For instance, how the PaCER project(s) you sponsored moved into additional phases, been shared more widely, informed healthcare policy/practice.
12. Are there aspects of the program delivery that could be improved upon? If so, please elaborate.
13. Is there anything else you’d like to share?

Demographic Questions (if you are comfortable answering)

1. How would you describe your gender identity?
2. How would you describe which age range you were in when you first sponsored a PaCER project?
   1. 18 years old – 29 years old
   2. 30 – 39 years old
   3. 40 – 49 years old
   4. 50 – 59 years old
   5. 60+ years old
3. How would you describe your field of research when you first sponsored a PaCER project?
4. How would you describe your ethnicity/cultural background?
