# Supplementary material for: Impact of a Health Research Training Program on Patient and Community Partners, and Researchers: A Qualitative Evaluation
Source: Health Expect. 2026 Jun 26;29(4):e70731. doi: 10.1111/hex.70731 (PMC13307345; doi:10.1111/hex.70731)
Supplement: Supplementary file 3 — Supporting File 3: hex70731‐sup‐0003‐coding_tree_PaCER_Evaluation. [file HEX-29-e70731-s002.docx]

| **Theme** | **Codes** | **Subcodes** |
| --- | --- | --- |
| **Seeing the value of the training program** | Motivations for joining PaCER | \| Academic recognition of Pacer \| \| --- \| \| Aligned with personal interests \| \| Bringing lived experience into research \| \| Building Patient Partner Network \| \| Certification \| \| Contributing to health policy \| \| Contributing to health research \| \| Empowerment \| \| Experiences as a Patient Partner \| \| Giving back to health community \| \| Increase in opportunities \| \| Making research accessible \| \| Professional development \| \| Supportive environment \| |
| **Learner readiness and capacity** | Challenges of PaCER | Barriers to engagement  Commitment to program for a year  Group dynamics  Lowered capacity due to health condition |
| **Diverse people and groups working together** | Clear understanding of role  Positive Experiences in PaCER | Building patient partner network  Group dynamics  Like minded individuals  Learning more about patient-oriented research  Academic rigor  Intense  Well supported |
| **Academic opportunities for learners and career growth** | Impacts PaCER post program | \| Advocacy \| \| --- \| \| Adopting a patient-oriented lens \| \| Aiding to awareness of program \| \| Building Patient Partner Network \| \| Changing misconceptions \| \| Conference presentations \| \| Experience navigating healthcare decision makers \| \| Experience navigating healthcare professionals \| \| Graduate studies \| \| Grant review opportunities \| \| improved reflexivity and positionality \| \| Increase in patient partner opportunities \| \| Increased employability \| \| Indigenous research methodology \| \| Long term impact \| \| more meaningful patient partner engagement \| \| Opening opportunities \| \| Patient-oriented research knowledge capacity increased \| \| Personal impact \| \| Presentation to governing health organization \| \| Professional development \| \| Publications \| \| Teaching better health system navigation \| \| Teaching better patient advocacy \| \| Transferable skills \| \| Working in a group/collaborative work \| |
