# Supplementary material for: Impact of a Health Research Training Program on Patient and Community Partners, and Researchers: A Qualitative Evaluation
Source: Health Expect. 2026 Jun 26;29(4):e70731. doi: 10.1111/hex.70731 (PMC13307345; doi:10.1111/hex.70731)
Supplement: Supplementary file 5 — Supporting File 5: hex70731‐sup‐0005‐GRIPP2_Short_form. [file HEX-29-e70731-s003.docx]

**Table 2**

GRIPP2 short form

| Section and topic | Item | Reported on page No |
| --- | --- | --- |
| 1: Aim | Report the aim of PPI in the study | 2 |
| 2: Methods | Provide a clear description of the methods used for PPI in the study | 2-5 |
| 3: Study results | Outcomes—Report the results of PPI in the study, including both positive and negative outcomes | 6-10 |
| 4: Discussion and conclusions | Outcomes—Comment on the extent to which PPI influenced the study overall. Describe positive and negative effects | 11-12 |
| 5: Reflections/critical perspective | Comment critically on the study, reflecting on the things that went well and those that did not, so others can learn from this experience | 12 |

PPI=patient and public involvement
